# Sulfur/carbon cathode material chemistry and morphology optimisation for lithium–sulfur batteries†

**DOI:** 10.1039/d4ra04740k

**Published:** 2024-09-26

**Authors:** Tayeba Safdar, Chun Huang

**Affiliations:** a Department of Materials Imperial College London London SW7 2AZ UK a.huang@imperial.ac.uk; b The Faraday Institution Quad One, Becquerel Ave, Harwell Campus Didcot OX11 0RA UK; c Research Complex at Harwell Rutherford Appleton Laboratory Didcot OX11 0FA UK

## Abstract

Lithium–sulfur batteries (LSBs) are a promising alternative to lithium-ion batteries because sulfur is highly abundant and exhibits a high theoretical capacity (1675 mA h g^−1^). However, polysulfide shuttle and other challenges have made it difficult for LSBs to be commercialised. Here, a sulfur/carbon (S/C) composite was synthesised and cathodes were fabricated *via* scalable melt diffusion and slurry casting methods. Carbon nanoparticles (C65) were used as both sulfur host and electrical additive. Various carbon ratios between the melt-diffusion step and cathode slurry formulation step were investigated. An increased amount of C65 in melt-diffusion led to increased structural heterogeneity in the cathodes, more prominent cracks, and a lower mechanical strength. The best performance was exhibited by a cathode where 10.5 wt% C65 (TC10.5) was melt-diffused and 24.5 wt% C65 was externally added to the slurry. An initial discharge capacity of ∼1500 mA h g^−1^ at 0.05C and 800 mA h g^−1^ at 0.1C was obtained with a capacity retention of ∼50% after 100 cycles. The improved electrochemical performance is rationalised as an increased number of C–S bonds in the composite material, optimum surface area, pore size and pore volume, and more homogeneous cathode microstructure in the TC10.5 cathode.

## Abstract 2

Lithium–sulfur batteries (LSBs) are one of promising alternative to lithium-ion batteries because sulfur is abundant and exhibits a high theoretical capacity (1675 mA h g^−1^).

## Graphical abstract

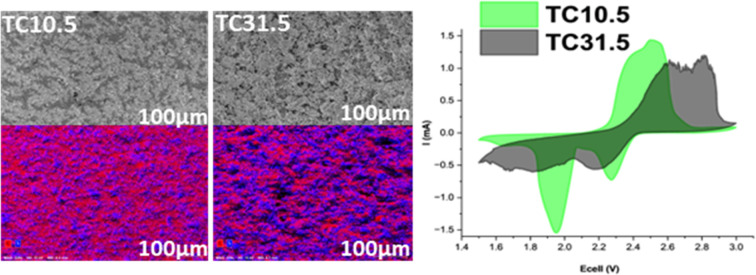

## 1. Introduction

Recent rises in average global temperatures and pollution have brought forth the consequences of abusing fossil fuels to meet the increasing energy demands. The severity of the damage and projected results (if fossil fuels are used at the current rate) have caused serious concerns about the sustainability of the planet. This has led to various investigations into alternate energy options and thus shifting the fuel focus to renewable methods to find sustainable solutions for the ever-increasing energy demands. As a consequence, batteries have gained immense popularity. Over the last decade the applications of batteries have attracted attention across many industries such as electric transportation and electrical energy storage from intermittent renewable sources. With lithium-ion batteries (LIBs) gradually reaching their physicochemical limits,^1^ the research focus is shifting towards next generation battery chemistries with the aim to develop batteries that are able to provide a balance between performance, cost and sustainability.^2^

Lithium–sulfur batteries (LSBs) have shown great promise due to their high energy density, low cost and natural abundance of sulfur element. Sulfur is one of the most abundant materials on earth. LSBs have a theoretical capacity of 1675 mA h g^−1^ and energy density of 2600 W h kg^−1^ as opposed to 300 mA h g^−1^ and 265 W h kg^−1^ for conventional LIBs with oxide-based cathodes.^3^ Conventional LSBs use liquid electrolytes, commonly comprised of Li salts and solvents to act as a charge transfer medium and ionic conductor.^4^ Theoretically LSBs provide an excellent solution to the current challenges of LIBs. However, LSBs are held back because they exhibit a poor capacity retention, short lifetime and large volume expansions (up to 22%).^4,5^ The presence and extent of these challenges are significantly increased due to the polysulfide (PS) shuttle mechanism causing a domino effect and significantly reducing the efficiency of the batteries.^6^

During the discharge cycle, Li metal anode is oxidised to from Li^+^ ions. These diffuse across the electrolyte to the cathode where sulfur, S_8_, is reduced to lithium sulfide (Li_2_S). The redox reactions of first discharge cycle can be summarised in four main stages.^7,8^ Stage 1 occurs during initial discharge process. The bonds between the sulfur rings are broken and long PS chains are formed of S_*x*_^2−^ (4 ≤ *x* ≤ 8). In the second stage long PS chains are disassociated from S_*x*_^2−^ (4 ≤ *x* ≤ 8) to S_*y*_^2−^ (1 ≤ *y* ≤ 3). In stage 3 (third phase of discharge cycle) S_3_^2−^ chains are reduced to S_2_^2−^. In the 4th stage, final stage of the discharge cycle, S_2_^2−^ is broken down to S^2−^. In the charge cycle, Li^+^ ions move back to anode whilst being reduced to Li metal by accepting electrons from the external circuit, Li_2_S is oxidised back to elemental sulfur by losing electrons. Sulfur will go through solid–liquid–solid phase changes during charging and discharging. When solid state S_8_ is discharged to a high order PS, it will dissolve in the electrolyte and react with Li anode to form low order PS,^9^ short PS chains are insoluble in liquid electrolyte. The diffusion of high and low order PS in and out of the electrolyte is what causes the shuttle effect.^3,10^ As S_8_ atoms are arranged in a cyclic ring, the bonds between the atoms can be associated and disassociated in various patterns. This instigates unwanted and complicated redox reactions, making it hard to exactly predict what type of PS will be formed.^11–13^

When high order PS reacts with Li anode, low order PS is formed and can be deposited on the Li anode and sulfur cathode. Thus, excessive solid electrolyte interface (SEI) is formed at the electrode interphase, restricting Li^+^ pathway and blocking access to sulfur active material,^14^ causing a non-uniform deposition of Li.^15^ As a result, SEI cracks into the electrolyte, continuing the cycle of reaction between Li anode, formation of new SEI and electrolyte components.^14,16,17^ Since Li^+^ pathways are constantly being blocked, damaged or changed, the battery performance after first few discharge cycles is significantly impacted. Battery then suffers from lowered ionic conductivity, volume expansion as the reaction chemistry can be unpredictable, shorter lifetime as well as corrosion and loss of active material.^18–21^

Many studies have focused on either eliminating or mitigating the shuttle effect. This can be achieved *via* adding PS inhibiting additives to liquid electrolyte or modifying electrode and separators structures by adding polar materials (*e.g.* O and N incorporated in polymers),^22–25^ adding functional groups and/or designing composites (*e.g.* mesoporous titanium nitride – sulfur),^26^ use of vulcanised polymer structures like polypropylene,^2,27^ single walled carbon nanotube coatings on separators,^28^ adding interlayers to prevent migration of PS to the Li anode,^29^ introducing various carbon structures like nanotubes, nanofibers, carbon aerogels^30–32^ to trap PS chains, *etc.* Solid-state electrolyte may also reduce the shuttling effect due to the lack of production of high order long chain PS, so side and unwanted reactions in conversion of S_8_ to Li_2_S may be reduced (depending on the reaction between solid-sate electrolyte, SEI formed and electrode).^2,33–35^

Carbon-based sulfur cathodes are being researched, more specifically focusing on the type of carbon and the role it plays. It can be used to enhance the electrical conductivity, act as a sulfur host or a part of a copolymer to suppress the shuttle effect. Carbon nanotubes (CNTs), graphene and porous carbons (macro- (>50 nm), meso- (2–50 nm)- and microporous (<2 nm)) are amongst the commonly used for sulfur carbon (S/C) cathode modification and optimisation. Materials such Super P, carbon black C65 and C45 are used in current LIBs and have desirable properties to be used as part of LSB cathodes.^36,37^ Their high surface area, mechanical properties, and good electrical conductivities possess a good solution to the insulation property of elemental sulfur. These conductive porous carbons are not part of active material and do not participate in the redox chemical reactions. Therefore, careful consideration is needed when deciding the amount used as less is preferable for an overall high energy density at the battery cell level.^37^ Due to this, there is always a trade-off between how much sulfur content a good S/C cathode should contain.^38^ Many studies vary sulfur wt% between 40 and 70 wt% (ref. 39–46) and achieve discharge capacities between ∼500–900 mA h g^−1^ with ∼50–70% capacity retention over 50–150 cycles. This paper focuses on utilising already existing carbon materials and investigating the balance between the carbon host material in the chemical composition of cathode composite material during synthesis and the electrical conductivity enhancer in the formulation of cathode suspension during slurry coating.

Currently, synthesis of S/C composites is achieved either *via* melt diffusion or solution-based methods. Melt diffusion is employed with the intention of trapping sulfur into the porous/fibrous carbon structure. It is usually carried out by mixing and heating sulfur and carbon powders. When heated, sulfur has a relatively low viscosity and high mobility, therefore making it easier to be loaded into carbon pores through capillary forces. In solution-based methods organic solvents like carbon disulfide (CS_2_), toluene, xylene or dimethyl sulfoxide are used to dissolve sulfur followed by drying the solution to obtain S/C powder composites. In other cases, thiosulfate or sulfides are mixed with carbon solution (such as graphene oxide) and reacted with an acid (such as hydrochloric acid) to form S/C composites which are dried to obtain as powders.^47–52^ The solution-based methods usually involve the use of toxic chemicals. Another approach is sulfur vapour deposition (SVD) where sulfur powder is evaporated in a furnace at temperatures of 300–600 °C under a certain atmosphere, commonly used argon, where the argon carrier gas helps the sulfur vapour travel forward to cooler part of the furnace so it can be deposited on the substrate (commonly used stainless steel, carbon structures or current collector).^53,54^ This technique can allow for binder free cathodes.^55^ However, SVD involve the use of sulfur vapour at elevated temperatures posing increased safety concerns. SVD also has limitations of involving the use of specialist equipment and being energy intensive.^52,56–60^ Based on these safety concerns and to eliminate the use to toxic chemicals as much as possible, this paper implements the use of dry melt-diffusion method without any toxic organic solvents to from S/C composites to make LSBs more sustainable for industrial applications.

## 2. Results and discussion

### 2.1. Morphology and mechanical properties

**Fig. 1:**
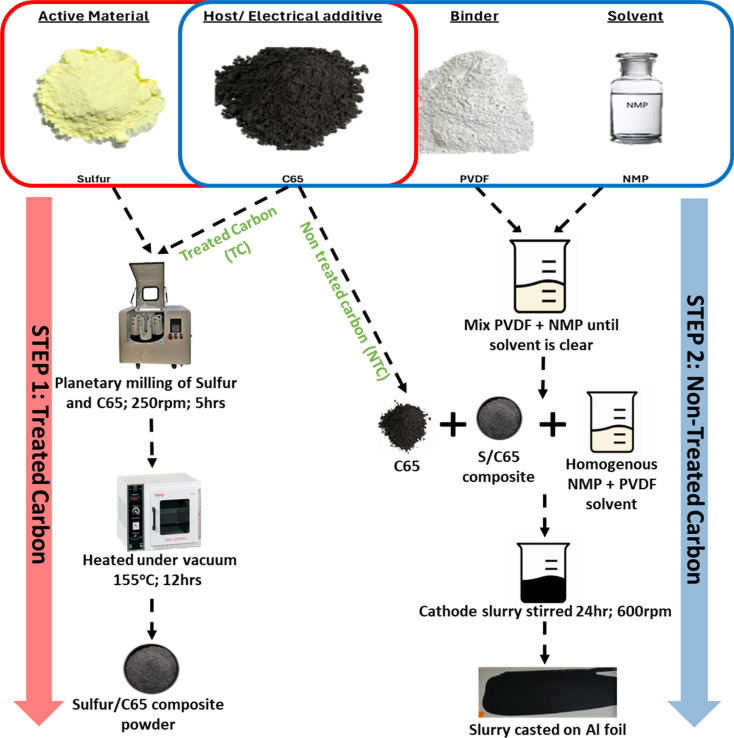
Schematic of cathode synthesis.

S/C composite was synthesised first *via* melt diffusion of sulfur and C65 to infuse sulfur onto the carbon structure. Cathodes were then fabricated by suspending the S/C composite with C65 in 1-methyl-2-pyrrolidinone (NMP) solvent to make a slurry followed by slurry coating, *i.e.* C65 was used both as a sulfur host in the composite material and as an electrical additive in the cathode structure, therefore C65 was added in two different stages. Fig. 1 shows a schematic of how the material synthesis and electrode fabrication were carried out.

The fabricated cathodes thickness was kept constant at 45 μm. This was achieved through doctor blade coating (70–55 μm) followed by calendaring. The total wt% of C65 was maintained constant at 35 wt% in each cathode while the proportion of treated carbon (TC, melt diffused carbon with sulfur) and non-treated carbon (NTC, externally added carbon in cathode slurry) was varied. Table 1 shows 5 different cathode configurations made with their respective notations, mass loading and density at the same thickness. The density of the cathode was increased from TC3.5 to TC31.5 with the amount of TC. Additionally, TC3.5 to TC17.5 exhibited a gradual increase in density and almost no change in mass loading, while steep increases in both mass loading and density were observed with TC24.5 and TC31.5. This was largely attributed to the cathode slurry properties and an increased amount of particle settling in higher TC slurries, leading to inhomogeneous casting of the slurry. High mass loadings (>2 mg cm^−2^) in S/C cathodes can cause fractures, delamination, enhanced shuttle effect and slow reaction kinetics,^61^ therefore many preliminary studies keep mass loadings low (<2 mg cm^−2^).^62^ For this study, all the mass loadings were kept below 3 mg cm^−2^ with the aim of first determining optimum cathode ratio between sulfur active material, carbon host and electrical additive. Once a standard cathode configuration is achieved, higher mass loadings can be investigated to overcome the stated challenges of poor mechanical properties and slow redox reactions.^63–66^

**Table 1:** Different cathode configurations, denoting the amount carbon that was treated (TC) in step 1 and non-treated carbon (NTC) added in step 2 and physical characteristics of different types of cathodes

Step 1: treated carbon (TC wt%)	Step 2: non-treated carbon (NTC wt%)	Cathode notation	Mass loading (mg cm^−2^)	Thickness (μm)	Density (mg cm^−3^)
3.5	31.5	TC3.5	1.20	45	477
10.5	24.5	TC10.5	1.23	45	495
17.5	17.5	TC17.5	1.23	45	504
24.5	10.5	TC24.5	2.35	45	946
31.5	3.5	TC31.5	2.67	47	1020

**Fig. 2:**
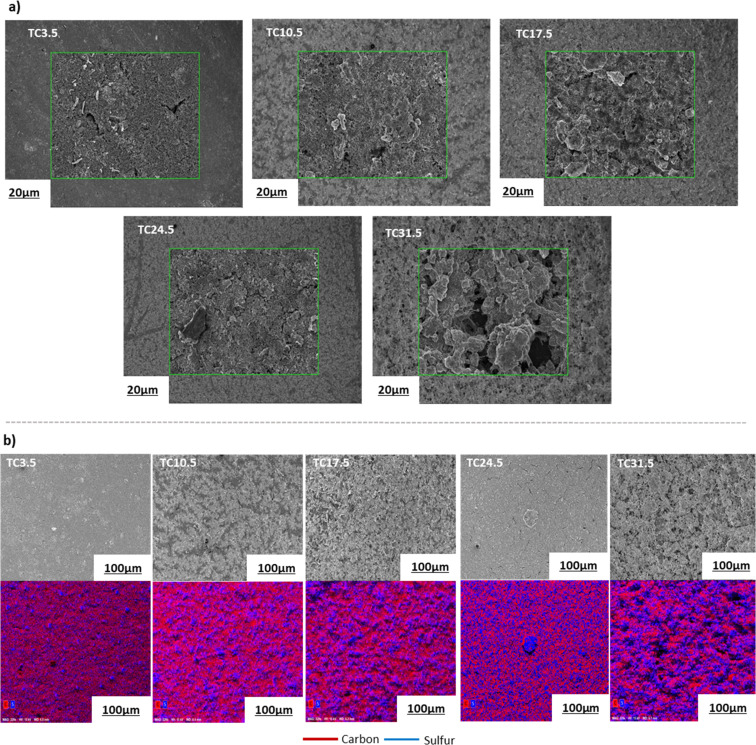
(a) SEM images of the magnified surface morphology of different types of cathodes; (b) overview SEM images and the corresponding EDX elemental mapping of the cathode surface morphology. Fig. S1 in ESI† shows SEM images of TC10.5 cathode film and *vs.* TC10.5 composite powder. Table ST1 in ESI† shows EDX mass and atomic% of all cathode samples.

Electrode microstructure and physical properties are heavily governed by the slurry suspension properties. Achieving a desirable suspension depends on many factors such as slurry composition, active material properties, type of conductive agent used, as well as casting and drying procedures.^67^ Scanning electron microscopy (SEM) images in Fig. 2a show that as the TC ratio increased, the particle distributions in the cathode microstructure became more uneven and the cracks became more prominent. TC31.5 exhibited the highest degree of physical defects with increased heterogeneous deposition of cathode composite material across the current collector. This may be because as the amount of C65 treated with sulfur increased, the cathode slurry became more unstable. Energy dispersive spectroscopy (EDX) mapping in Fig. 2b displays pockets of sulfur in various sizes which were more prominent in higher TC cathodes. TC24.5 and TC31.5 had the highest amount of sulfur insulation pockets. These were formed because some sulfur that was loaded on carbon recrystallised during the melt diffusion process.

Fig. S2–S5 in the ESI† shows the adsorption and desorption isotherms and surface areas measured by the Brunauer, Emmett and Teller (BET) method for all TC cathodes, pure sulfur, pristine C65, and heat treated C65. Table 2 summaries the specific surface area (SSA_BET_), pore volume and average pore size conducted on the different TC cathode composites, pristine C65, heated C65 (HC65; heated at 155 °C for 12 h) and pristine sulfur. SSA_BET_ of C65 was measured to be 65 m^2^ g^−1^ which matched with literature and industrial values.^68^ A decrease in SSA_BET_ was observed for HC65 compared with C65, suggesting a small change in carbon microstructural arrangement after heating. Both C65 and HC65 exhibited a type II isotherm which is typical of microporous (5–50 nm pores) samples. Pure sulfur as expected had the lowest SSA_BET_ and pore volume, confirming the non-porous characteristics. TC31.5 exhibited the highest SSA_BET_ (0.49 m^2^ g^−1^), pore size (28 nm), and pore volume (0.0041 cm^3^ g^−3^) among the different TC cathodes. An ideal S/C cathode should exhibit a high enough surface area to promote and favour S_8_ reduction to Li_2_S and be porous enough to trap insoluble PS chains whilst allowing diffusion of long PS chains to establish favourable reaction kinetics.^69^ These properties are best displayed by TC10.5 where a high SSA_BET_ of 0.45 m^2^ g^−1^ was combined with smaller pore width and volume (14 nm and 0.0028 cm^3^ g^−1^).

**Table 2:** TC cathode BET specific surface area, adsorption pore volume (BJH, *p*/*p*^o^ = 0.99) and average pore width

	Specific surface area (SSA_BET_) (m^2^ g^−1^)	Adsorption pore volume (cm^3^ g^−1^) (*p*/*p*^o^ = 0.99)	Average pore width (nm)
Pristine C65	64.90	0.1897	12
HC65	60.00	0.1873	12
TC3.5	0.37	0.0018	9
TC10.5	0.45	0.0028	14
TC17.5	0.32	0.0021	23
TC24.5	0.32	0.0020	14
TC31.5	0.49	0.0041	28
Pure sulfur	0.16	0.0006	10

### 2.2. Physicochemical properties

**Fig. 3:**
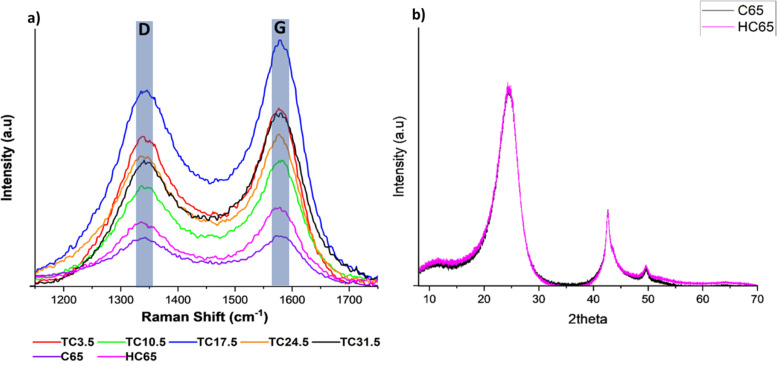
(a) Raman D and G peaks for different TC cathodes, HC65 and pristine C65; (b) XRD spectrum of pristine C65 and HC65.

Raman spectroscopy was carried out to further understand bond changes that occurred in C65 across the different TC samples. D band is the result of out of plane vibrations attributed to the structural defects. The D band peak occurring at 1347 ± 3 cm^−1^ represents disorder in C–C bonds and denotes defects.^70–73^ The D band peak was observed at 1347 cm^−1^ instead of 1350 cm^−1^ for conventional carbon materials such as graphite because C65 contains an increased number of sp^3^ carbon atoms. The bond length of sp^2^ C–C (∼1.33 Å) is shorter than C–C sp^3^ bond length (∼1.54 Å), longer bond length contributes to a lower wavelength in Raman spectra.^74–77^ The G band peak occurring at 1595 ± 3 cm^−1^ represents the first order scattering of sp^2^ carbon atoms and denotes graphitic bonds. The scattering is caused by in-plane vibrations of sp^2^ hybridised carbon atoms.^68,78^ Raman spectroscopy was carried out on all TC samples along with 100% C65 heat treated under the same conditions (Fig. 3a). Higher *I*_D_/*I*_G_ ratio correlates to more structural defects present on carbon sites. Since C65 is mainly amorphous carbon, it is expected to have a relatively high *I*_D_/*I*_G_ value where the presence sp^3^ carbon is dominant over graphitic sp^2^ carbon. Defects can be categorised into intrinsic and extrinsic. Intrinsic defects are formed by sp^3^ hybrid carbon atoms.^79,80^ Extrinsic defects are formed because of electronegative heterogenous atoms combined with carbon, leading to defects being induced into the cathode composite.^81,82^ This is usually achieved *via* introducing functional groups (such as N, O,^83–85^ nickel nanoparticles,^86^ metal oxides, metal sulfides, metal carbides and metal nitrides),^87–92^ surface modification or in this case carbon sulfur (C–S) bonds (leading to strong chemical adsorption of PS chains through increased surface polarity).

The *I*_D_/*I*_G_ ratio was 0.97 and 0.81 for pristine C65 and HC65, respectively. This suggests that heat treating C65 at various temperatures for prolonged periods of time can reduce the number of defects present on the carbon surface and form some graphitic bonds.^93,94^Fig. 3b shows X-ray diffraction (XRD) patterns of pristine C65 and HC65 (Fig. S6† shows the XRD patterns of all TC cathodes). Three main peaks were at 24.2°, 42.5° and 49.6°. The first two were the response of [002] and [011] planes, respectively.^95^ The peak occurring at 49.6° could correspond 10*L* which would cover single peak representing [100] and [101] planes. As there were no separate peaks for [100] and [101], this confirms the presence of turbostratic structures in C65.^96^ Since XRD shows crystalline phases, the XRD patterns here only show the crystalline structure with ordered bonds for both samples. Nevertheless, the peaks are consistent with most carbon black materials where the carbon atom layer is not fully stacked.^96^

Table 3 shows all TC samples have *I*_D_/*I*_G_ ratio higher than HC65, indicating the reaction and formation of bonds between sulfur and carbon most likely at the defective sites of C65 during the melt diffusion process. The ratio amongst TC samples is a combination of naturally occurring intrinsic C65 defects along with extrinsic defects induced from C–S bonds. The presence of extrinsic defects promote redox reactions and chemical adsorption of PS chains, whereas intrinsic defects promote physical adsorption of PS chains.^97^ Therefore, higher ratio means more extrinsic defects being induced *via* C–S bonds. In some cases, sulfur can sit in these sites rather than forming homogenous C–S bonds through out.^98^ TC10.5 had the highest ratio of 0.88, and the *I*_D_/*I*_G_ ratio exhibited a steady decline after TC10.5 to TC31.5. As the amount of C65 heat treated with sulfur increased, the number of C–S bonds formed increased until TC10.5, hence, the high ratio of TC10.5 reflected the highest number of extrinsic defects (promoting redox kinetics as well as trapping PS chains). After TC10.5, the number of C–S bonds formed did not change, rather the intrinsic defects from C65 were filled/blocked by crystallised sulfur (sulfur insulation pockets shown in EDX mapping in Fig. 2b). Since more defect sites were blocked with increasing TC, the *I*_D_/*I*_G_ ratio decreased as TC increased from 10.5 to 31.5. Thus, it can be inferred that S/C cathodes with more defects and C–S bonds are more favourable in the lithium–sulfur battery configuration to trap more PS chains and improve the LSB performance.

**Table 3:** *I*_D_/*I*_G_ ratio calculated from D and G peaks intensities from Raman spectra

Sample	*I*_D_/*I*_G_
Pristine C65	0.97
HC65	0.81
TC3.5	0.84
TC10.5	0.88
TC17.5	0.86
TC24.5	0.85
TC31.5	0.83

**Fig. 4:**
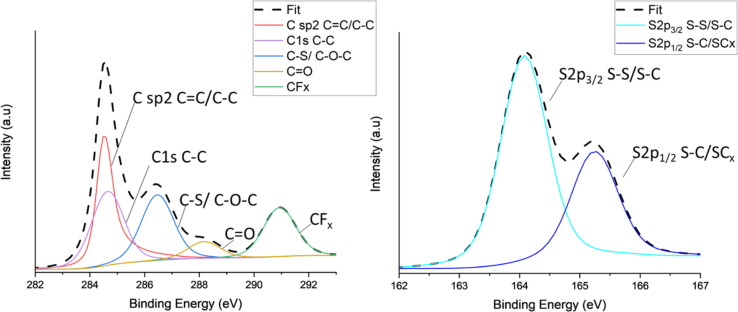
XPS peaks for TC10.5 with (a) C 1s; (b) high resolution S 2p spectra.

X-ray photoelectron spectroscopy (XPS) was carried out on all TC cathodes to study and evaluate the changes in bonds. Fig. 4 shows the spectra of carbon C 1s (Fig. 4a) and sulfur S 2p (Fig. 4b) for TC10.5, the profiles were deconvoluted into relevant Gaussian–Lorentzian peaks. The C 1s and S 2p spectra for the other TC cathodes are shown in Fig. S9a and b.† As C65 is a zero-dimensional amorphous soft carbon with turbostratic structure, C65 carbon would consist of a significant amount of sp^2^ carbon peak at around 284.5 eV, representing C–C bonds present due to the aromatic clusters of C65.^99,100^ The C–C peak corresponding to C 1s bond was also in the same position but a lower amount. The C–O–C/C–S peak was observed at 286.5 eV. The presence of O may be due to the presence of defects and the melt diffusion process which was performed under weak vacuum conditions. The peak at 290.7 eV indicates the presence of CF_*x*_ due to the bond between C65 and polyvinylidene fluoride (PVDF) binder in the cathode. The strong appearance of the CF_*x*_ peak indicates that drying duration and increases in drying temperature can lead to PVDF binder to rise from the bulk of the cathode to the surface.^101^ According to Pauling scale, the electronegativities of carbon and sulfur are in very close proximity to each other (2.55 and 2.58 respectively), making it extremely difficult to distinguish between carbon, sulfur and various carbon sulfides (CS_*x*_ and CS_2_).^102^ Therefore, it is difficult to track the exact changes in carbon–sulfur bond that are present. Fig. 4b shows the sulfur 2p spectra consisted of two main peaks of 2p_3/2_ at 164 eV and 2p_1/2_ at 165.1 eV. The 164 eV peak corresponded to elemental terminal sulfur bonds of S–S or C–S, where sulfur could be linked to carbon rings or an oxygen atom.^103,104^ The O1s spectra for all TC cathodes in Fig. S10† show no presence of O–S bonds, therefore, the sulfur 2p_1/2_ peak can be regarded as CS species, this aligns well with the C–S peak in C1s in Fig. 4a.^105^ The sulfur 2p_1/2_ peak corresponds to C–S–C bond with sulfur being the central atom.^106^ TC10.5 also exhibited the higher atomic% of C–S/C–O–C bond in C 1s spectra, confirming an increased presence of C–S bonds and defects in S/C cathode.

### 2.3. Electrochemical properties

**Fig. 5:**
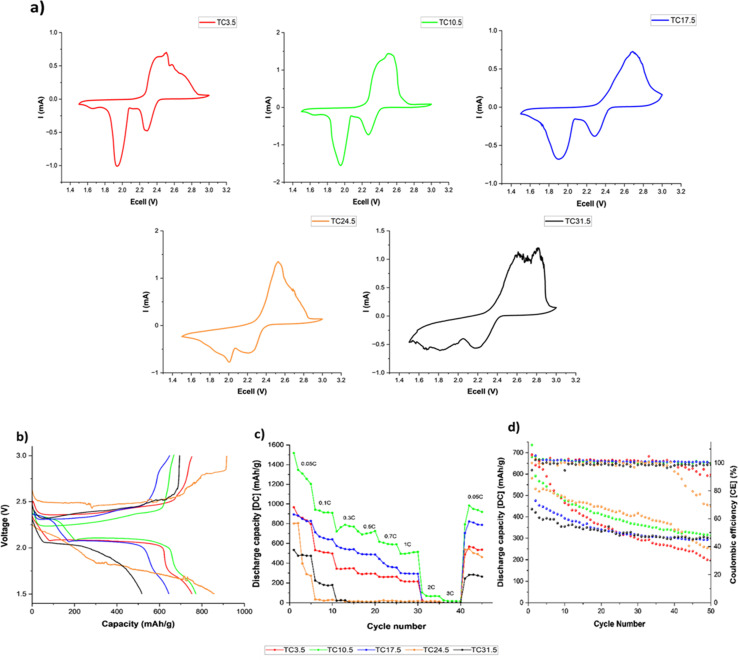
(a) Cyclic voltammograms at 0.05 mV s^−1^ scan rate before cycling; (b) initial charge discharge profiles at 0.5C; (c) discharge capacity during rate capability testing; (d) discharge capacity and coulombic efficiency at 0.1C during 50 cycles. All tests were performed for all TC cathodes in coin cell configurations at room temperature.

Fig. 5a shows the cyclic voltammogram of all TC cathodes at 0.05 mV s^−1^ scan rate before cycling in coin cell configurations. Two distinct redox peaks (2.3 and 1.9 V) during discharge were present for lower TC (TC3.5–17.5), representing the two-step reduction to S_8_ to Li_2_S.^99,107,108^Fig. 5b shows the charge discharge profiles of TC24.5 and TC31.5 at 0.5C. The peak at 2.3 V in the cyclic voltammogram was attributed to the first plateau of long chain PS_*x*_ chains formation (4 < *x*< 8) in the discharge profile, and the peak at 1.9 V in the cyclic voltammogram corresponded to the second plateau in the discharge curve for the conversion of long chain PS chains to short chain PS_*x*_ (1 < *x*< 3). Fig. 5a shows the two reduction peaks in cyclic voltammograms for TC24.5 and TC31.5 were broad and exhibited small shifts from the expected peak positions. This could be the combined result of slow reaction kinetics, where two step reduction of S_8_ is slow and inefficient. The broad peak could also represent the simultaneous reduction of S_8_ to Li_2_S. In such cases, the obtained cyclic voltammogram peaks are not well defined and/or are quite broad.^109–111^Fig. 5b shows both TC24.5 and TC31.5 profiles are noisy, indicating increased resistance within the cell arising from PS chains and their side reactions.

Fig. 5c shows lower TC cathodes (3.5, 10.5, and 17.5) exhibited better rate capabilities as the (dis)charge rates increased, TC10.5 exhibited the best rate capability. As the amount of externally added C65 in lower TC cathodes was greater, the cathode structure provided a better electron percolation network compared with higher TC samples where more C65 was added and heat treated in the first synthesis step, leading to inhomogeneous slurry and particle deposition during the second electrode coating step.^112^ The poor capacity and rate capability of higher TC cathodes were because of the increased mechanical defects (large cracks) and uneven TC particles distributions in the higher TC cathode microstructure, with the most sulfur insulation pockets in TC31.5 which slowed down redox reactions,^98^ as shown in the SEM and EDX images in Fig. 2. Increased inhomogeneity in microstructure led to limited access to active materials, decreased utilisation of active material, poor electrical percolation network, and hindered Li^+^ diffusion pathways.

Fig. 5d shows discharge capacity retention after 50 cycles at 0.1C. Higher TCs had better capacity retention ∼70%, however, their starting capacities were also lower (600 and 450 mA h g^−1^ for TC24.5 and TC31.5, respectively). TC10.5 had a high starting discharge capacity of ∼750 mA h g^−1^ and had a capacity retention of 46%. Fig. 5c shows the rate capability testing and when the C rate returned to 0.05C, TC10.5 exhibited a discharge capacity of 1000 mA h g^−1^ whilst TC24.5 and 31.5 displayed a discharge capacity of ∼550 and ∼300 mA h g^−1^, respectively. Overall, TC10.5 displayed consistent performance at different C rates and the highest discharge capacity at the end of 50 cycles. This is also supported in stability test where TC3.5, TC10.5 and TC31.5 continued cycling to complete 100 cycles (0.1C, shown in Fig. S11†). TC10.5 displayed a stable discharge capacity of ∼300 mA h g^−1^ at the end of 100 cycles whereas TC31.5 and TC3.5 exhibit a decrease in discharge capacity of ∼230 mA h g^−1^ and ∼100 mA h g^−1^, respectively, at the end of 100 cycles.^113–115^

Across literature numerous methods and configurations of sulfur cathodes have been explored with the aim of improving lithium sulfur battery performance. Many studies aim to achieve this *via* a combination of cathode optimisation, electrolyte additives, separator modification and/or different anode configurations. Popular cathode studies involve carbon nanotubes as sulfur host. Li *et al.*^116^ reported sulfur/carbon nanotubes as one of their cathodes, exhibiting ∼55% capacity after ∼100 cycles at 0.5C. These results are similar to the results exhibited by TC10.5 (Fig. S11†). Similarly, Han *et al.*^117^ reported multiwalled carbon nanotube/sulfur cathodes exhibiting capacity retentions between ∼31–40% after 100 cycles along with poor rate capability performance of 400 mA h g^−1^ at 0.1C as opposed to ∼600 mA h g^−1^ of TC10.5. Marangon *et al.*^118^ exhibited a capacity loss of ∼40% at ∼0.1C over 100 cycles. Qiao *et al.*^119^ report their sulfur/carbon nanotube cathode of 60–70 wt% sulfur with a capacity retention of 40–50% after 100 cycles at 0.2C. Though there are studies that demonstrate higher discharge capacities and better long-term stability^120–122^ a lot of variations are present across literature, *e.g.* using different ratios in heat treatment (such as sulfur : carbon wt. ratios of 3 : 7, 5 : 5, 4 : 6, 7 : 3 *etc*) without explaining the effect^66,123–125^ and almost all studies across literature with similar cathode design use at least 10 wt% as binder content in the cathode. TC10.5 has shown comparable performance of similar capacity retention after 100 cycles with only 5 wt% binder to reduce insulation/non-conductive elements in the cathode. Furthermore, all the capacities reported in this paper were calculated based on the total mass of the cathode including the binder and carbon which more realistically represents the performance in practical batteries. The aims of this study was to (i) find the optimum ratio of carbon black that can undergo a simple heat treatment with sulfur to form the sulfur/carbon composite; (ii) demonstrate successful performance of all the cathodes in this study with the use of only 5 wt% binder to reduce insulation/non-conductive elements in the cathode; and (iii) demonstrate synthesis of sulfur/carbon cathode utilising the slurry casting method that is compatible to the existing lithium ion battery fabrication in industry.

**Fig. 6:**
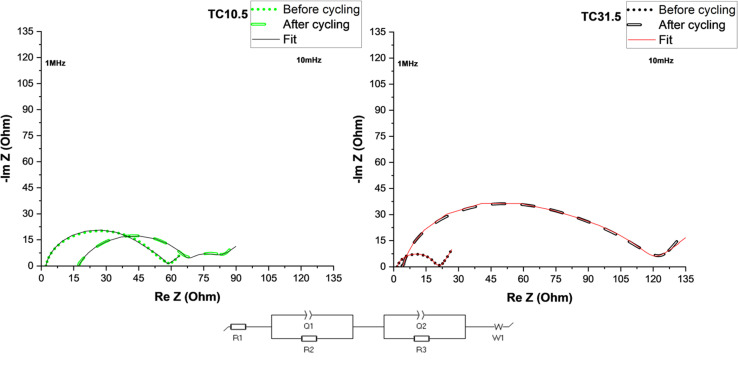
EIS plots of TC10.5 and 31.5 before and after 50 cycles at 0.1C.

Fig. 6 shows electrochemical impedance spectroscopy (EIS) plots of TC10.5 and TC31.5 with the equivalent circuit used to fit the data. *R*1 corresponds to ohmic resistances of electrolyte, current collector and cell connections, *R*2 is the combined interfacial resistances (of Li^+^ diffusion) between the electrode surface and electrolyte, *Q*1 is the distributed capacitance of sulfur and lithium electrode surface layers, *R*3 is PS charge transfer resistance on the sulfur electrode, *Q*2 is the distributed double layer capacitance of cathode surface pores and *W*1 represents Warburg diffusion of Li^+^ ions.^126–130^

Table 4 displays detailed values of resistances before and after cycling for TC10.5 and TC31.5. Changes in *R*1 after cycling represent the amount of soluble PS chains present in the electrolyte.^131^ After 50 cycles, more soluble long chain PS were present in TC10.5 than TC31.5. Similarly interfacial resistance *R*2 is an indication of the amount of insoluble short chain PS that is deposited onto the electrodes. In TC10.5, *R*2 decreased from 11.9 to 10.9 Ω, highlighting the depletion in deposition of insoluble short chain PS at the interfaces, and thus, improving the Li diffusion and interfacial wettability.^132^*R*2 for TC31.5 was tripled after cycling. Therefore, TC31.5 had a strong PS shuttle effect where access to sulfur active material and Li metal anode was blocked *via* constant formation of excessive unwanted SEI layer. Due to the presence of extrinsic induced defects and strong C–S bonds in TC10.5, there was a smaller increase in the charge transfer resistance, *R*3, before and after cycling when compared with TC31.5 where the *R*3 was almost 30 times greater after cycling. This aligned well with the hypothesis that the defective sites were being blocked by sulfur crystals. Electronic conductivity, from Table ST2,† was seen to increase with TC. Highest electronic conductivity was exhibited by TC31.5. Based on sulfur insulation pockets being predominantly present in higher TC, this was the unexpected result. Upon further inspection it was found that when C65 was heated at 155 °C for 12 hours, an increase in electronic conductivity was observed (almost 5 times higher). This clearly explains the unexpected trend of electronic conductivity increasing with TC, as the amount of HC65 was higher. In higher TC, though sulfur blocked pores and insulated the defects from being utilised, movement of electrons was faster due to network channels formed between heated C65 and C65 added in step 2 (shown in Fig. 1). Thus, a trade-off was required between enhancing electronic conductivity and the utilisation of sulfur active material and mitigation of PS shuttle to enhance battery performance.

**Table 4:** EIS resistance and capacitance values before and after cycling for TC10.5 and TC31.5. Pristine TC10.5 *a*1 = 0.77 and *a*2 = 0.91; TC10.5 after 50 cycles *a*1 = 0.92 and *a*2 = 0.75; pristine TC31.5 *a*1 = 0.88 and *a*2 = 0.70; TC31.5 after 50 cycles *a*1 = 0.95 and *a*2 = 0.69

	TC10.5		TC31.3	
	Pristine	After 50 cycles	Pristine	After 50 cycles
*R*1 (Ω)	2.15	17.16	1.64	4.08
*R*2 (Ω)	11.92	10.86	17.40	53.50
*R*3 (Ω)	43.85	51.14	2.11	61.50
*Q*1 (Fs^(*a*1-1)^)	3.81 × 10^−4^	4.76 × 10^−3^	6.10 × 10^−6^	5.74 × 10^−6^
*Q*2 (Fs^(*a*2-1)^)	4.01 × 10^−6^	1.88 × 10^−5^	6.37 × 10^−3^	1.69 × 10^−4^

Overall, TC10.5 had a medium surface area (0.45 m^2^ g^−1^), pore size (14 nm), and pore volume (0.0028 cm^3^ g^−1^) among all the TC cathodes from the BET results, which exhibited a high enough surface area to promote S_8_ reduction to Li_2_S and was porous enough to allow diffusion of long PS chains and trap insoluble PS chains. TC10.5 also had the highest *I*_D_/*I*_G_ ratio of 0.88 from the Raman results and the highest atomic% of C/S bonds from the XPS results, indicating the presence of more C–S bonds increased chemical adsorption of PS chains, aided redox reactions at higher C rates and increased rate capability of LSBs.

## 3. Conclusions

Multiple ratios of C65 heat treated with sulfur were investigated to establish the optimum amount of carbon that was added in the composite cathode material during melt diffusion synthesis (step 1) and added in the cathode slurry during electrode coating (step 2) for LSBs. The homogeneity of the cathode slurry decreased with an increasing proportion of treated carbon in step 1. Higher TC (from 17.5 to 31.5) suspensions were more unstable, leading to inhomogeneous deposition of cathode particles in the resulting cathode microstructure. TC17.5 or higher led to denser cathodes with elevated mechanical damage (defined cracks) thus directly impacting the electrochemical performance of the cells. TC10.5 provided the best electrochemical response of an initial discharge capacity of ∼800 mA h g^−1^ at 0.05C, best rate capability and low impedance. This may be because TC10.5 exhibited the highest *I*_D_/*I*_G_ ratio of 0.88 from Raman data and the highest atomic% of C/S bonds from XPS results, indicating the highest number of C/S bonds introduced from melt diffusion which promoted sulfur redox reactions and chemical adsorption of PS chains to reduce the PS shuttle effect while higher TC above 10.5 created more isolated sulfur pockets which were recrystallised during melt diffusion. TC10.5 also exhibited a medium, more optimum surface area, pore size and pore volume to facilitate long PS chain diffusion and trap insoluble short PS chains. This study demonstrated that good performing S/C composite cathodes were synthesised and fabricated by using commercially available starting materials and scalable methods. The dry melt diffusion method does not require the use of toxic solvents such as CS_2_, benzene or toluene in the conventional solution-based C/S composite synthesis method. The dry melt diffusion process also used a lower heating temperature of 155 °C (as opposed to some other studies of melt diffusion and that conduct melt diffusion and sulfur vapour deposition at 300–600 °C), making the processing more sustainable. Therefore, the synthesis process in this paper closely resembles current industrial slurry casting and cathode synthesis technology for lithium ion batteries and thus has the potential for scaling up using existing casting equipment.

## 4. Experimental

### 4.1 Cathode fabrication

60 wt% sulfur and varying ratios of C65 were ground in a planetary ball mill in zirconium jars with 10 mm zirconium milling balls, with ball to powder ratio being 1 : 1 (v : v) at 250 rpm for 5 hours. To prevent oxidation of sulfur (due to the heat generated during mixing) the milling programme was set to run for 5 minutes followed by a rest period of 10 minutes to allow for adequate cooling of the powder. This is supported by the XRD data shown in Fig. S12.† Where no change in crystal structure was observed between the two samples. No change in structure was observed between powder that was milled in room atmosphere and one that was pack in airtight jar under Ar atmosphere. The milled powder was heat treated for 12 hours at 155 °C under vacuum conditions to make S/C composite (step 1). To make the cathodes, 5 wt% PVDF powder and non-heat treated C65 of different wt% were suspended homogeneously in 1-methyl-2-pyrrolidinone (NMP) solution first, and the S/C composite powder from step 1 was added. 5 wt% PVDF was used instead of report 10–8 wt% to reduce the insulation components in the battery. The slurry was cast onto aluminium foils and dried under the fume hood overnight then dried under vacuum at 60 °C for 1 hour. The dried cathodes were calendared (to reduce the thickness by 25–28%, ± 3%) before being cut into 16 mm discs. All the cathodes had an overall composition of S : C65 : PVDF = 60 : 35 : 5 (wt%). Cathode thickness was controlled at 45 μm.

### 4.2 Physical and chemical characterisation

Cathode morphology was studied *via* SEM on Zeiss Sigma 300 at 5 keV, this was combined with energy dispersive X-rays (EDX) (15 keV) to study the distribution of sulfur and carbon on cathode surface. XPS was utilised to study the changes in C/S bonds using a Thermo Scientific K-alpha X-ray photoelectron Spectrometer. Measurements were conducted on cathode discs with 35 eV pass energy for high resolution C 1s and S 2p spectra, and Thermo Avantage software was used for data analysis. BET analysis (conducted in autosorb iQ3) was used to determine the specific surface area, Barrett, Joyner and Halenda (BJH) method was used to determine the pore size distribution and pore volume. All TC samples were degassed at 25 °C for 15 hours prior to conducting the measurements and 100% C65 samples were degassed at 300 °C for 12 hours. XRD (conducted on Bruker D2) was used to study the changes in crystal structure of the cathodes. Measurements were conducted between 5–70° with a step size of 0.01°. Raman study was conducted on a Reinshaw instrument utilising 532 nm excitation laser.

### 4.3 Electrochemistry

Electrolyte was synthesised in an Ar glovebox by dissolving 1 M of lithium bis(trifluoromethanesulfonyl)imide (LiTFSI) and 0.8 M of lithium nitrate in 1 : 1 (v : v) 1,2 dioxlane and 1,2dimethoxyethane (DOL : DME) solvents. Both lithium salts were dried at 120 °C under vacuum before being transferred to glovebox. Both DME and DOL solvents were dried by placing molecular sieves (4 Å pore diameter) for 3 days and then filtered using PFTE syringe tips. Prior to use, molecular sieves were dried at 220 °C for 3 days and then transferred to glovebox. 16 mm diameter lithium chips were used as the anode, 19 mm diameter Celgard polypropylene 2400 was used as separator and 80 μL liquid electrolyte was used to assemble each CR2032 coin cell. All coin cells were rested overnight after assembly before carrying out electrochemical testing at room temperature. Galvanostatic charge and discharge and rate capability testing were performed on Arbin LBT20084. All the galvanostatic testing was carried out between the voltage range of 1.5–3 V. Different C rates of 0.05, 0.1, 0.3, 0.5, 0.7, 1, 2, 3 and 0.05C were evaluated. At each C rate the cell was (dis)charged 5 times in the order of the rates listed. All the cells were tested for 50 cycles at 0.1C. Cyclic voltammetry (CV) was conducted on pristine cells condition on Biologic BCS-128 at 0.05 mV s^−1^ scan rate. Electrochemical Impedance spectroscopy (EIS) was carried out in frequency range of 1 MHz–10 mHz at 10 mV sinus amplitude at open circuit voltage (∼2.2 V) on Biologic SP-300. Electronic conductivity of the cathode discs was measured using 2 point probe. DC voltages of 0.02, 0.06, 0.08, 0.12, 0.16 and 0.18 V were applied to the cathode which was sandwiched between two stainless steel electrodes to achieve an electrode blocking assembly. The current response at each voltage was recorded between 1–3 hours (or until a plateau was achieved). All measurements were carried at 25 °C.

## Data availability

The data supporting this article have been included as part of the ESI.†

## Conflicts of interest

There are no conflicts to declare.

## Supplementary Material

RA-014-D4RA04740K-s001
