# Supplementary material for: Sulfur/carbon cathode material chemistry and morphology optimisation for lithium–sulfur batteries
Source: RSC Adv. 2024 Sep 26;14(42):30743–55. doi: 10.1039/d4ra04740k (PMC11425154; doi:10.1039/d4ra04740k)
Supplement: RA-014-D4RA04740K-s001 [file RA-014-D4RA04740K-s001.pdf]

Supplementary Information (SI) for RSC Advances.
This journal is © The Royal Society of Chemistry 2024

Supporting Information

Sulfur/Carbon Cathode Material Chemistry and Morphology Optimisation for Lithium-Sulfur Batteries

Tayeba Safdar[a,b] and Chun Huang*[a,b,c]

[a] T. Safdar, Dr C. Huang
Department of Materials
Imperial College London
London SW7 2AZ, UK; E-mail: a.huang@imperial.ac.uk.
[b] T. Safdar, Dr C. Huang
The Faraday Institution
Quad one, Becquerel Ave, Harwell Campus, Didcot, OX11 0RA, UK
[C] Dr C. Huang
Research Complex at Harwell
Rutherford Appleton Laboratory
Didcot, OX11 0FA, UK

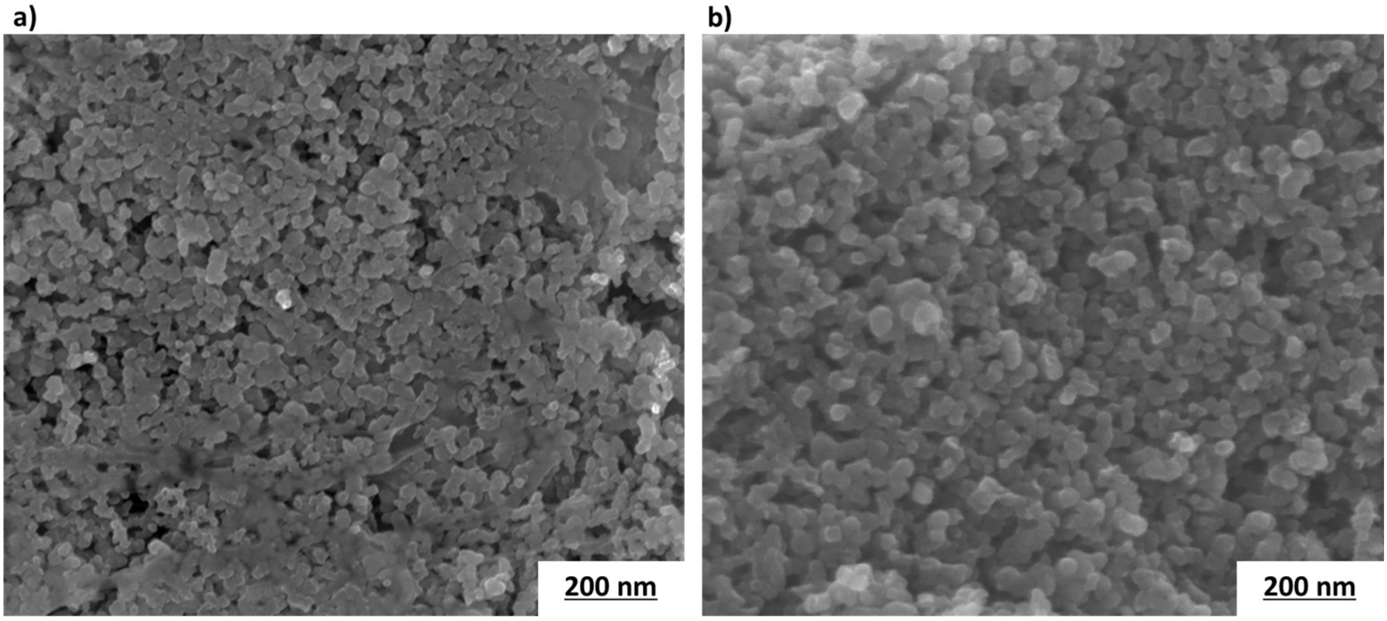

Figure S1. : a) TC10.5 cathode surface SEM image at 200nm; b) TC10.5 S/C composite powder surface SEM at 200nm

Sample	Mass concentration % (± 40 – 60%)	Atomic concentration %(± 40 – 60%)	Mass concentration %(± 53 – 60%)	Atomic concentration %(± 53 – 60%)

	Sulfur		Carbon	
**SC10**	35.4	17.0	64.6	83.0
**SC30**	26.9	12.1	73.1	87.9
**SC50**	23.1	10.1	76.9	89.9
**SC70**	34.4	16.4	65.6	83.6
**SC90**	35.7	17.2	64.3	82.8

Table ST1: EDX atomic and mass % across different cathode samples

BET Isotherms

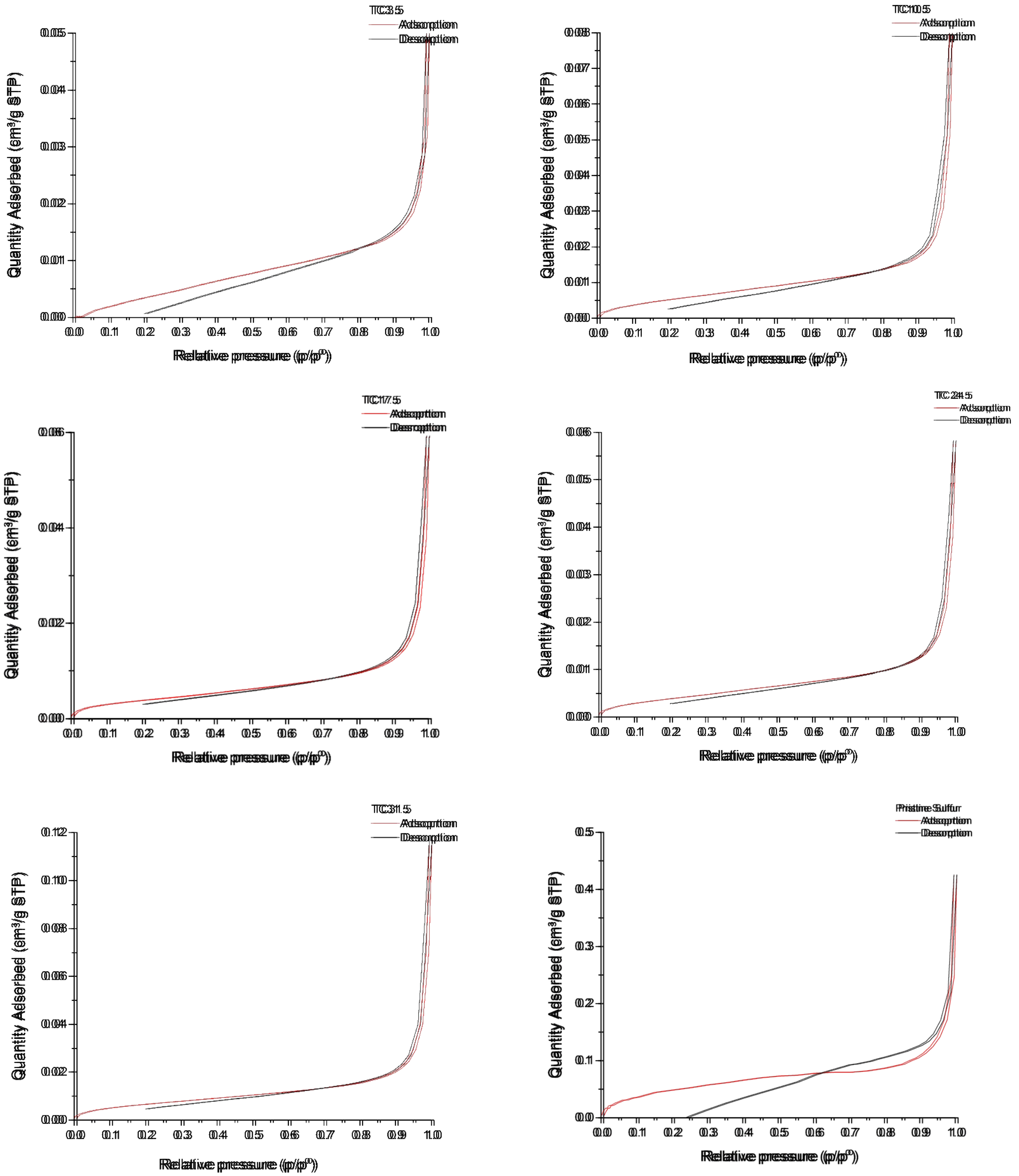

Figure S2: Adsorption and desorption isotherms for all TC sample and pristine sulfur

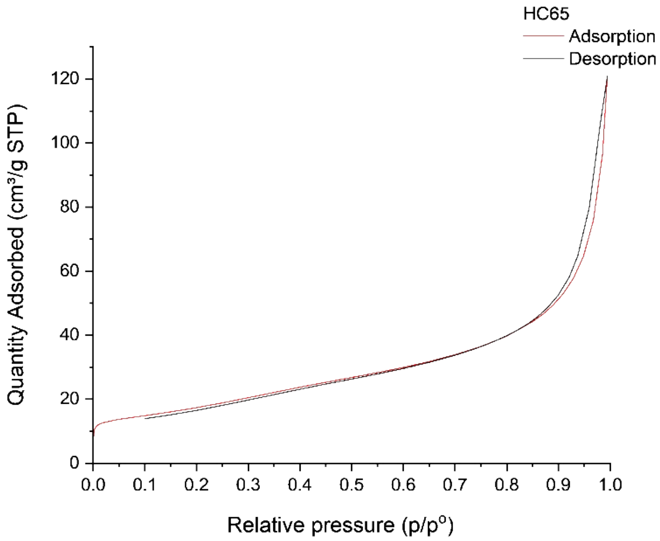

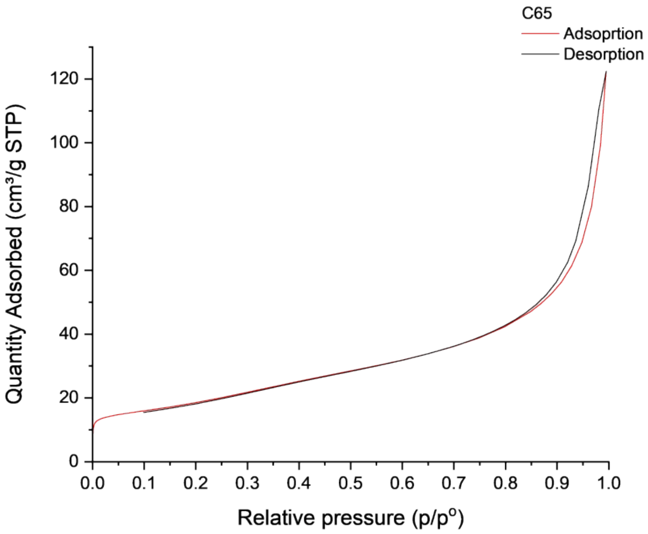

Figure S3: Adsorption and desorption isotherm for heated C65 and pristine C65

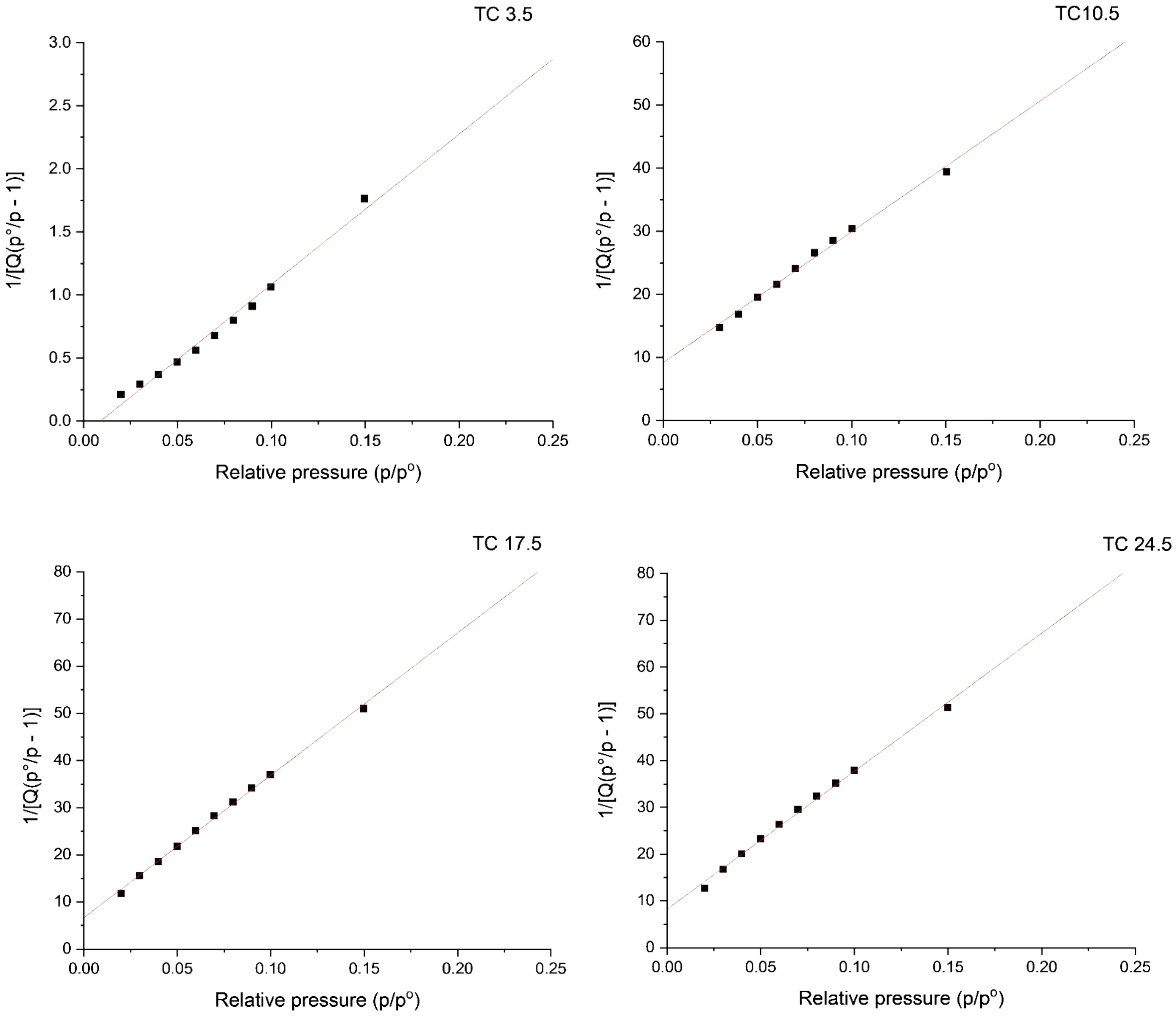

Figure S4: BET surface area plots for TC3.5, 10.5, 17.5 and 24.5

BET surface area plot

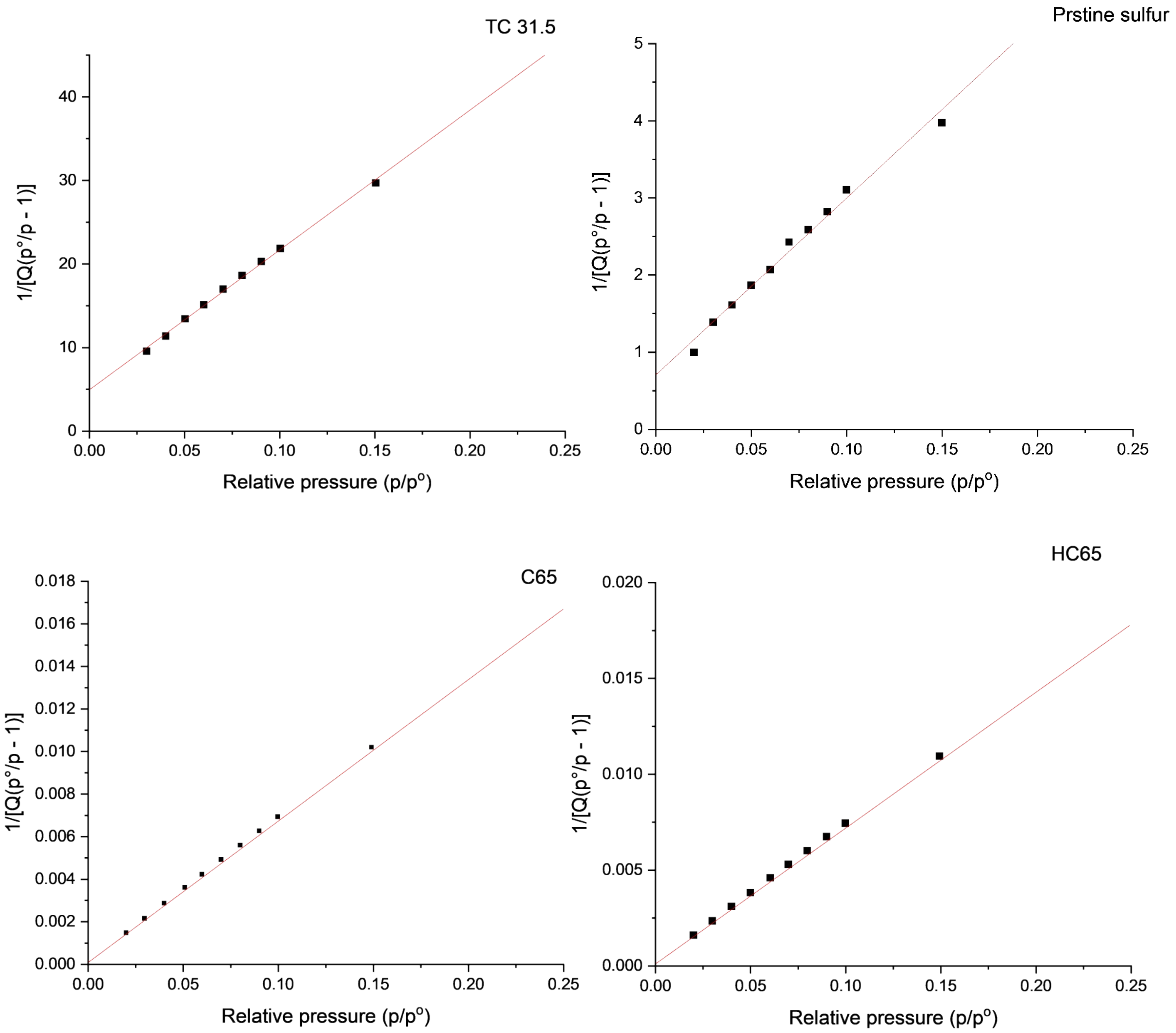

Figure S5: BET surface area plots for TC31.5, pristine sulfur, pristine C65 and HC65

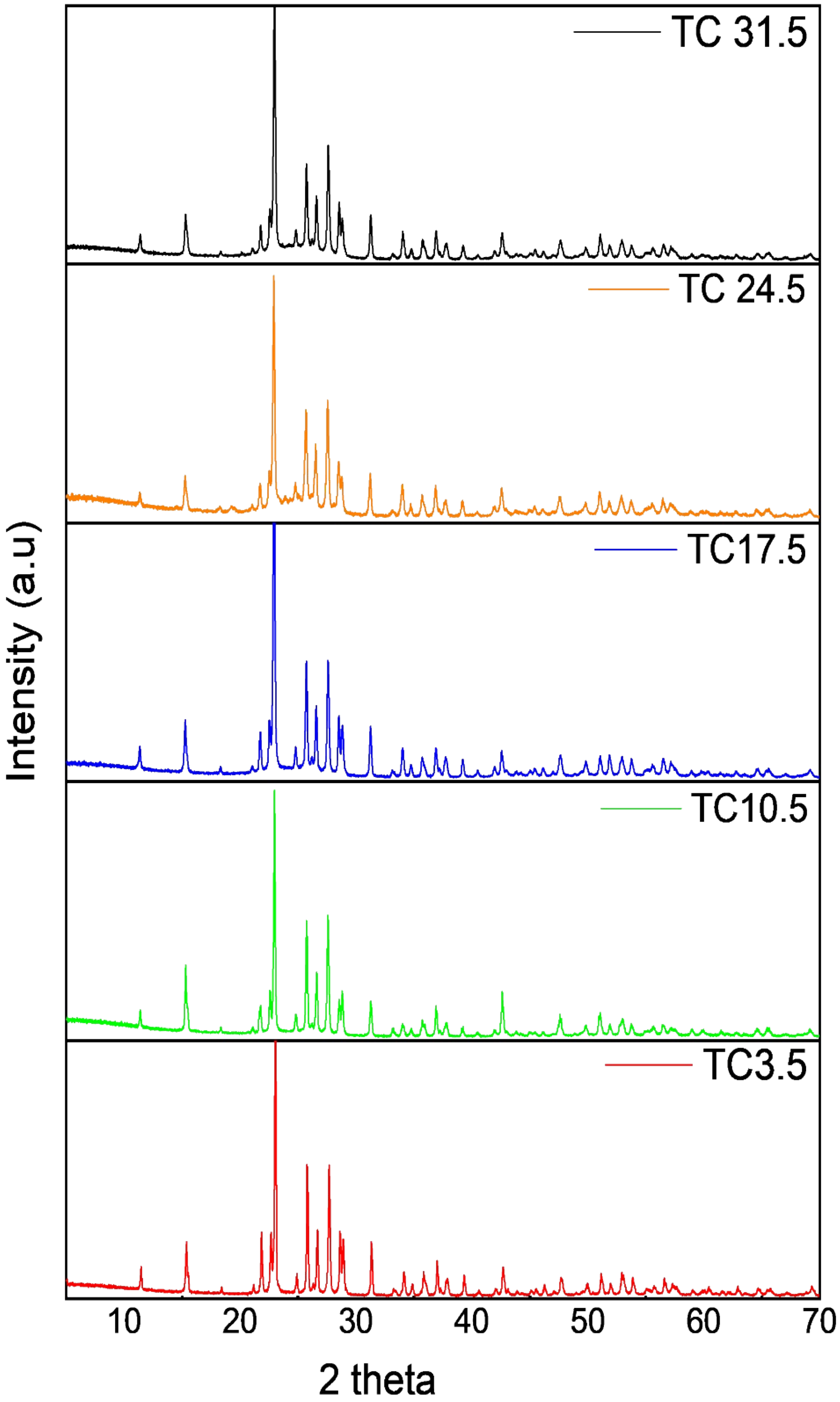

Figure S6: XRD pattern of TC cathode films

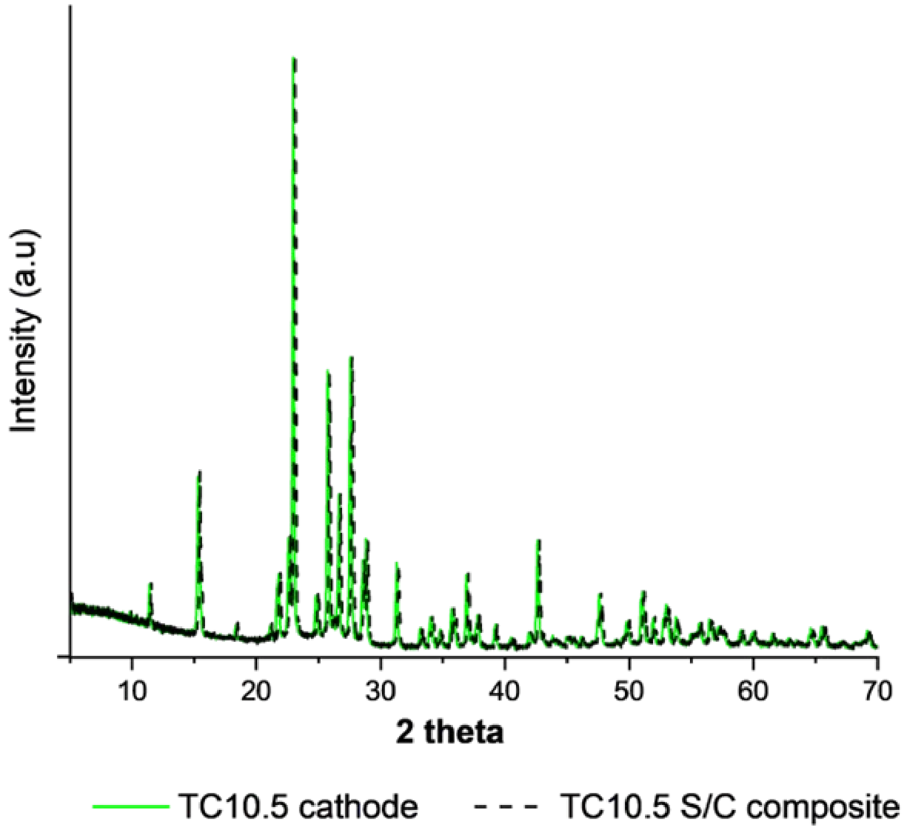

Figure S7: XRD pattern of TC10.5 cathode film with TC10.5 S/C composite

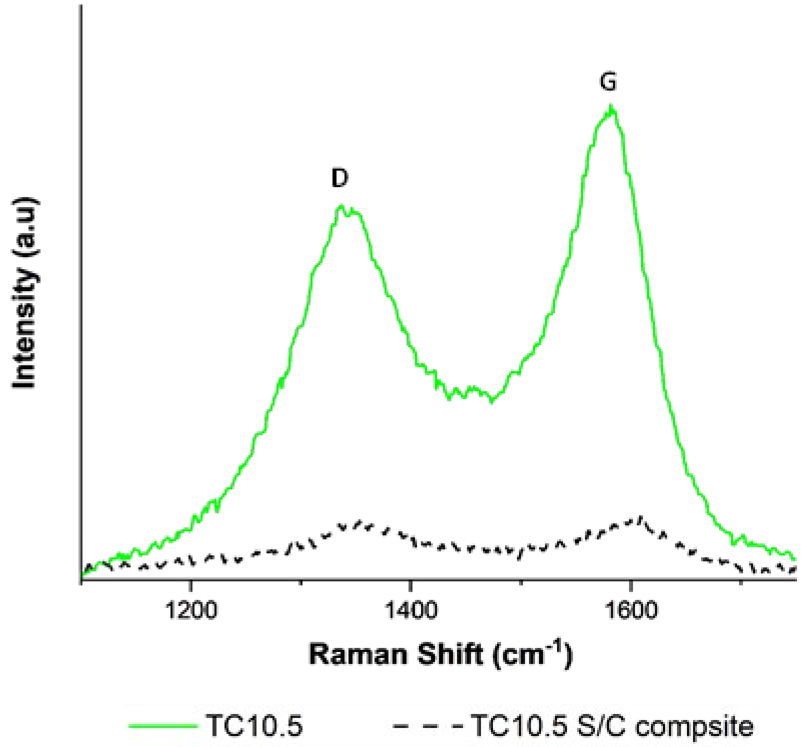

Figure S8: Raman D and G peaks pattern of TC10.5 cathode film with TC10.5 S/C composite

XPS C1s and Sp2 Spectra

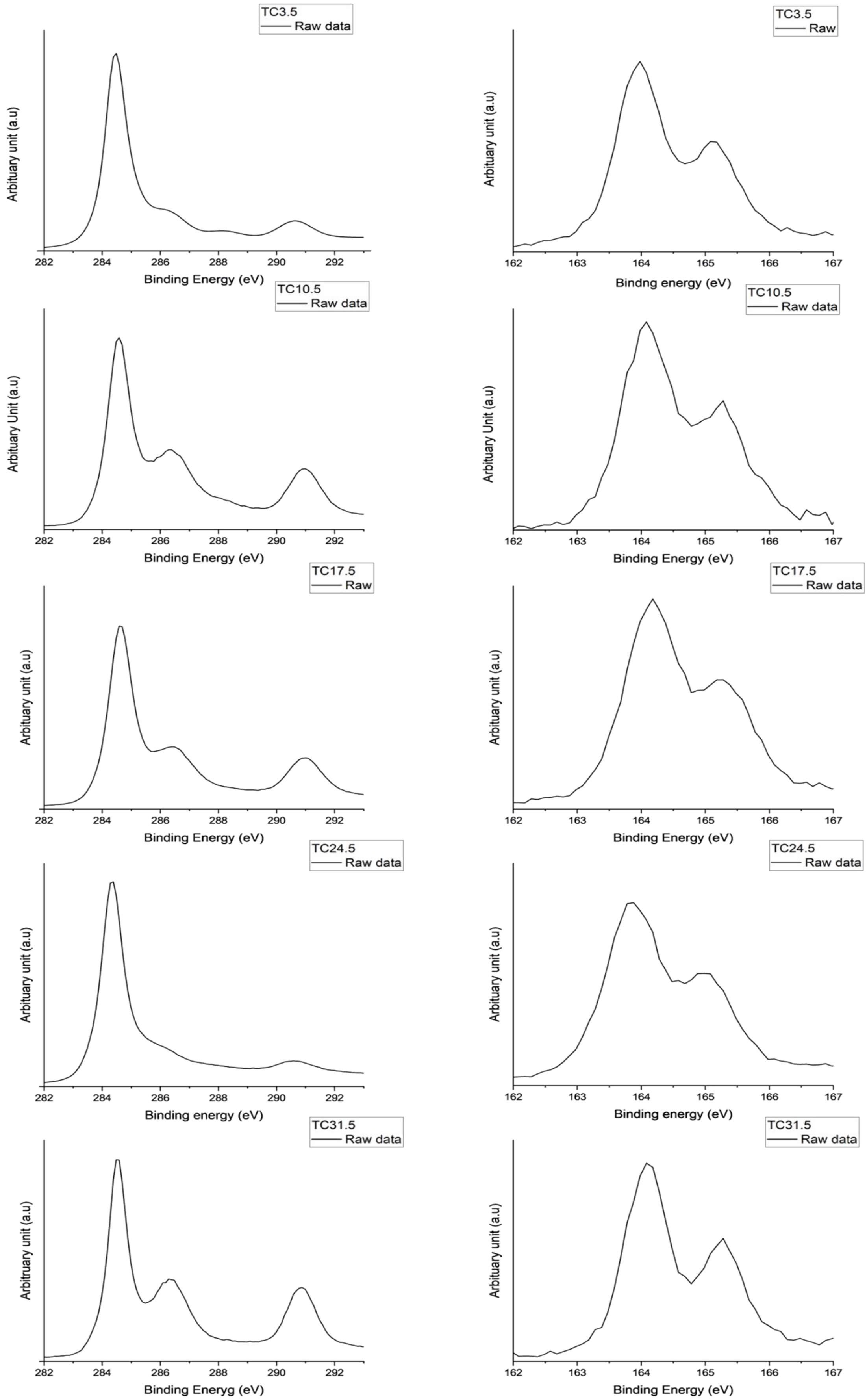

Figure S9a: C1s and S2p raw spectra for TC3.5, 10.5, 17.5, 24.5 and 31.5

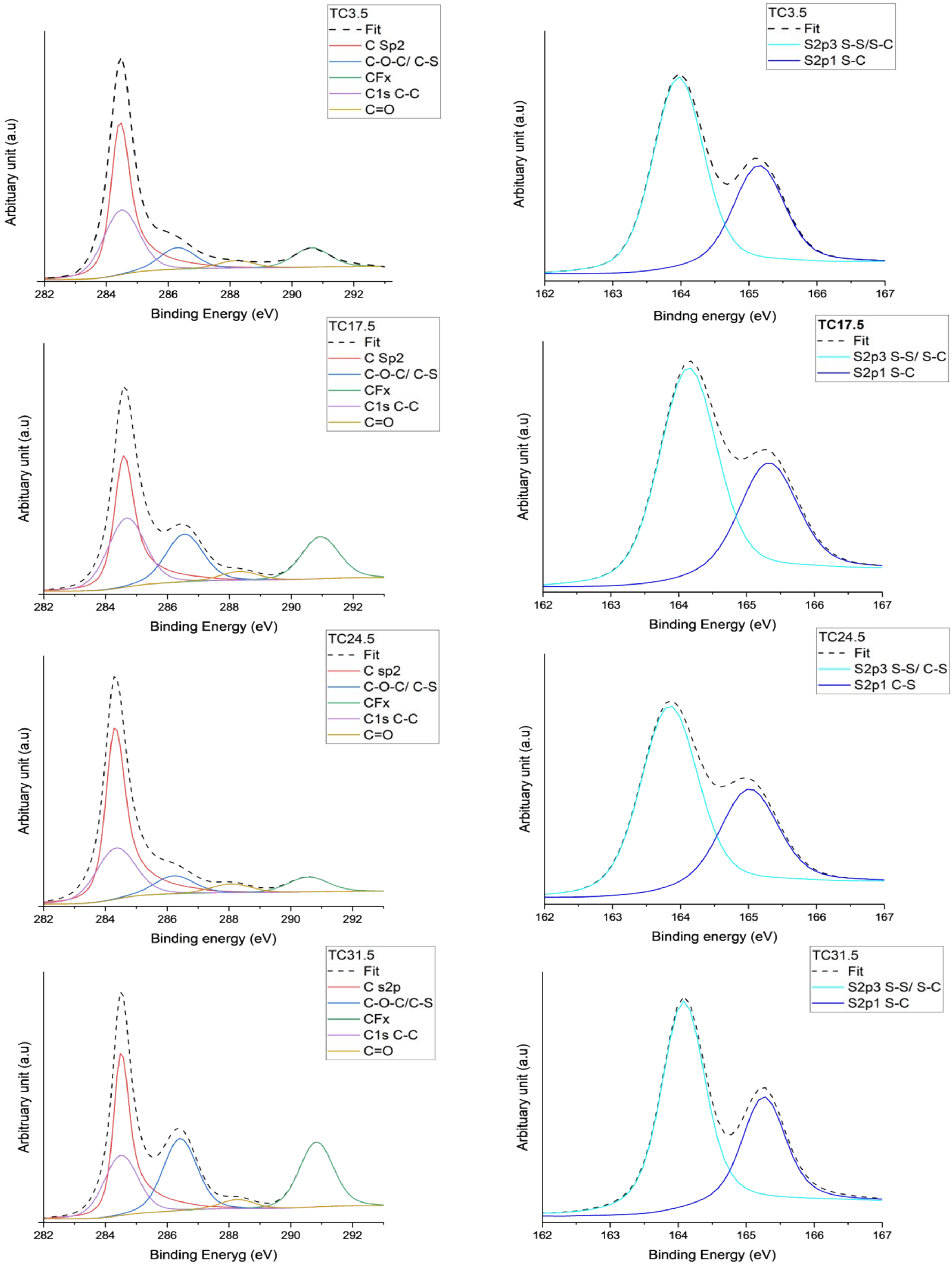

Figure S9b: C1s and S2p fitted spectra for TC3.5, 10.5, 17.5, 24.5 and 31.5

XPS O1s spectrum

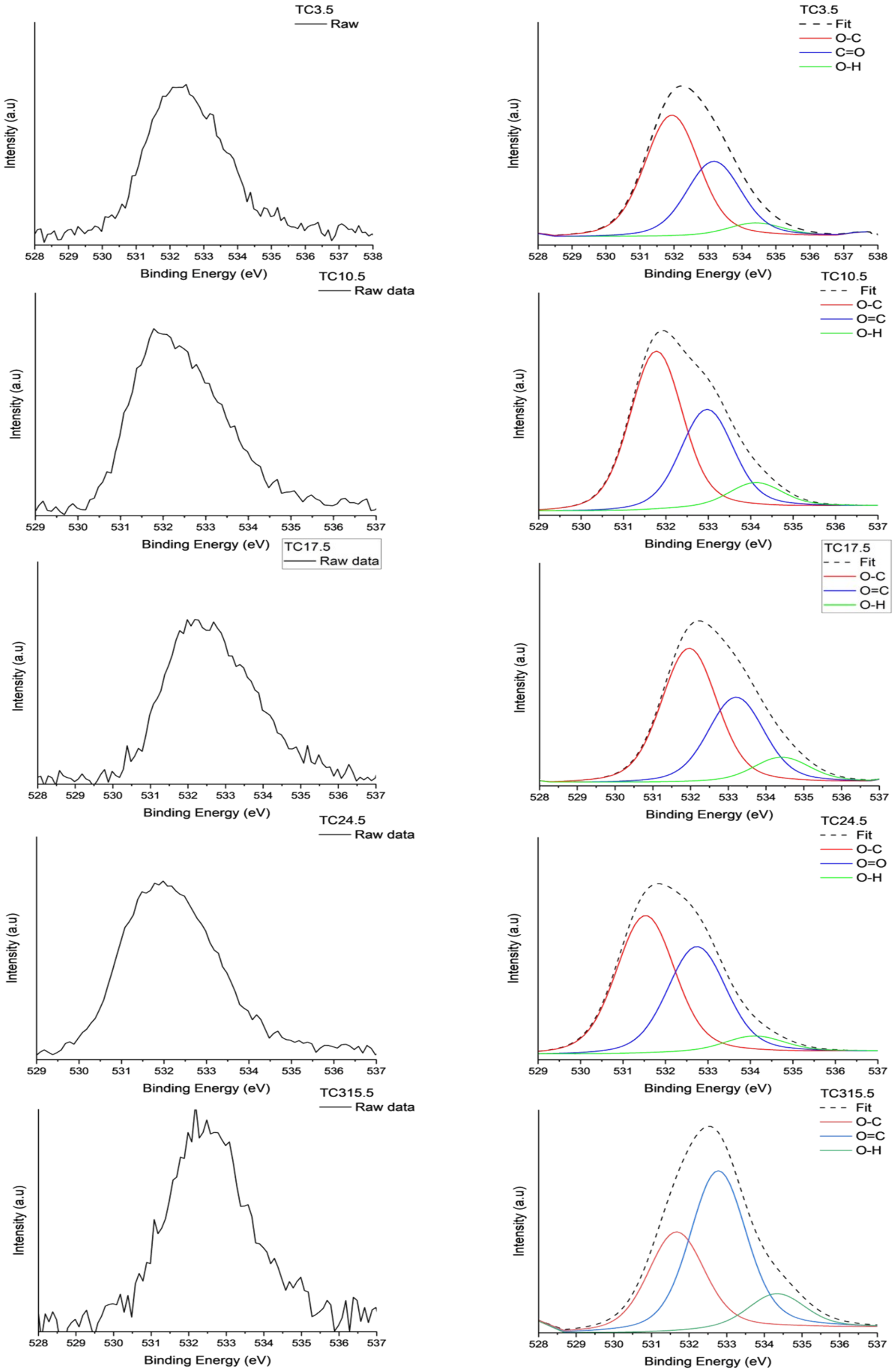

Figure S10: O1s raw and fitted spectra for all TC samples

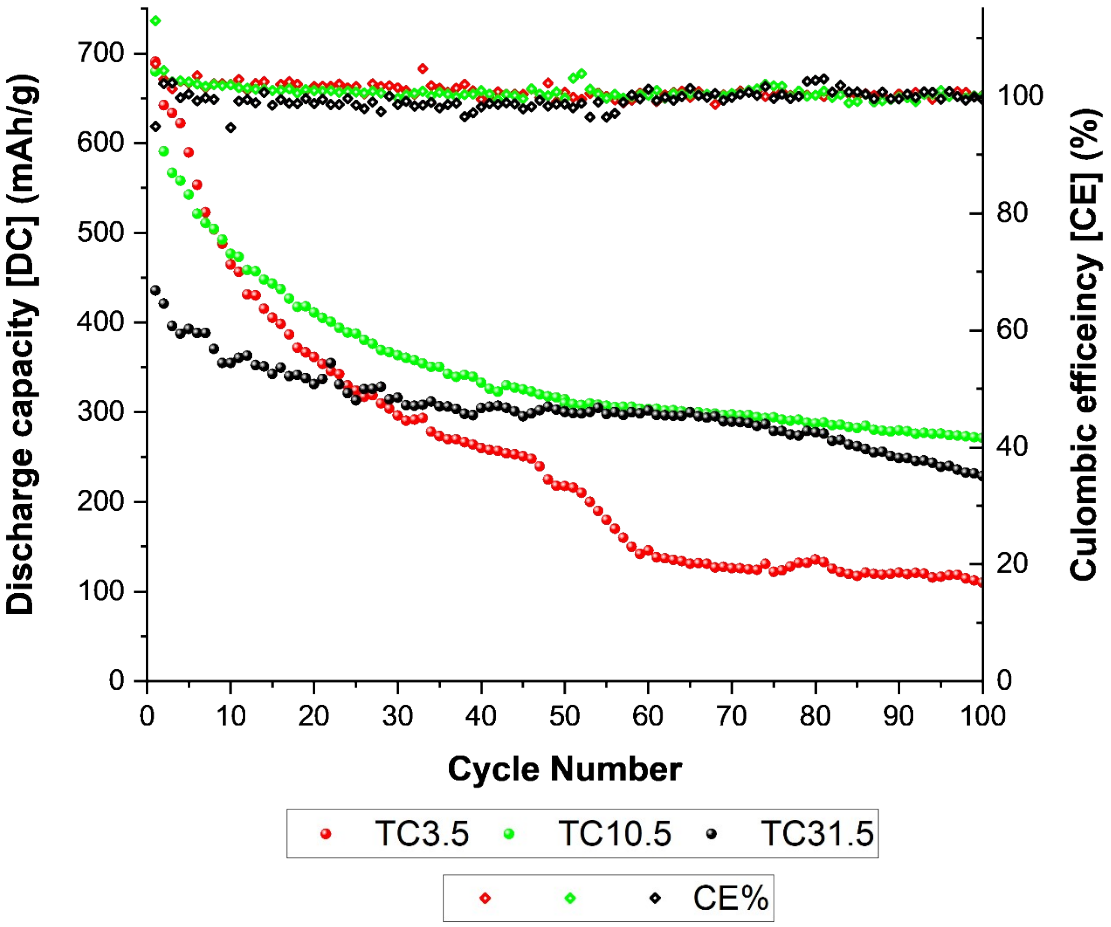

Figure S11: Discharge capacity and coulombic efficiency after 100 cycles

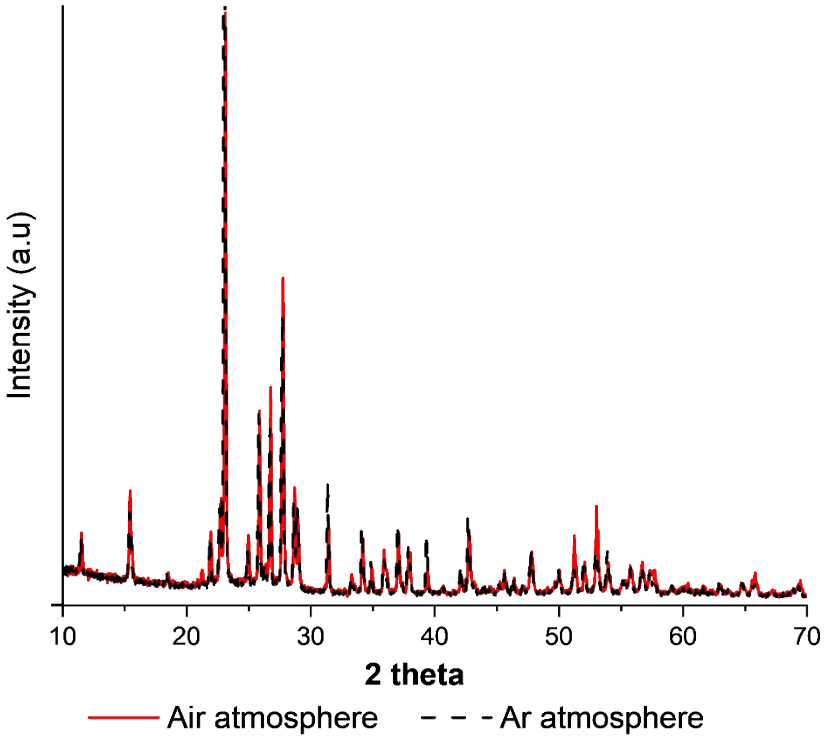

Figure S12: XRD pattern of TC composite milled in Ar and air atmosphere

Table ST2: Electronic conductivities of All TC cathode samples. C65 carbon black and heated C65 carbon black

Sample	σ = (1/R_{eff})*(d/A) (S/m)
**TC3.5**	1.77E-03
**TC10.5**	2.15E-03
**TC17.5**	3.00E-03
**TC24.5**	3.08E-03
**TC31.5**	4.34E-03
**HC65**	5.10E-01
**Pristine C65**	1.08E-01
